# Case Report: Reversible chorea arising from severe vitamin B12 deficiency due to autoimmune gastritis: a comprehensive case study

**DOI:** 10.3389/fnut.2025.1590837

**Published:** 2025-08-21

**Authors:** Meifang Yang, Yifan Geng, Zhiren Chen, Zixuan Zhou, Wenjuan Huang, Weiwei Chen, Xia Zhang

**Affiliations:** ^1^Department of Neurology, Xuzhou Central Hospital, Xuzhou Clinical School of Xuzhou Medical University, Xuzhou School of Clinical Medicine of Nanjing Medical University, Xuzhou, Jiangsu, China; ^2^Southeast University Affiliated Xuzhou Central Hospital, Xuzhou, Jiangsu, China; ^3^Department of Neurology, Xuzhou Clinical College, Xuzhou Medical University, Xuzhou, Jiangsu, China

**Keywords:** vitamin B12 deficiency, chorea, autoimmune gastritis, 18F-FDG PET/CT, movement disorders

## Abstract

Cognitive impairment, ocular neuropathy, sensorimotor polyneuropathy, and subacute combined spinal cord degeneration can all result from a common illness called vitamin B12 insufficiency. With regard to extrapyramidal movement disorders, it is rare, frequently misdiagnosed, and under recognized, which postpones timely treatment. A case study of a 66-year-old man with acute-onset and reversible choreoathetoid symptoms is presented in this publication. A significant vitamin B12 deficiency and an abnormal hypermetabolism in the basal ganglia region were detected by 18F-FDG PET/CT. Remarkably, the patient’s dyskinesia disappeared as soon as vitamin B12 replacement therapy was administered. The patient’s etiology was identified as a mix of vitamin B12 malabsorption and inadequate intake from autoimmune-related gastritis. In order to achieve the best potential therapeutic results, this case highlights the importance of timely and correct diagnosis, timely treatment of vitamin B12 deficiency based on its underlying etiology, and careful research of multiple etiologies.

## Introduction

Chorea is a hyperkinetic movement disorder characterized by involuntary, uncontrolled, and non-patterned motions that vary in speed, timing, and direction. It manifests as sudden, intermittent choreiform movements that affect any part of the body, mainly the distal limbs but also the face and trunk, and has the capacity to transfer across several body parts. The illness may be caused by autoimmune diseases, metabolic imbalances, certain drugs and hormones, hereditary neurodegenerative disorders, or structural damage to deep brain structures from a variety of causes. Acute chorea must be evaluated for metabolic variables, hypoxia, vascular disorders, infections, or systemic inflammation before pharmacological or toxic reasons are considered. Although electrolyte abnormalities and hypoglycemia rank among the most significant causes, vitamin B12 insufficiency is a fairly uncommon one. In addition to a number of uncommon symptoms such autonomic dysfunction, involuntary movements, vision impairment, epilepsy, and cognitive-emotional abnormalities, a deficiency in vitamin B12 can result in complex neurological symptoms, primarily peripheral neuropathy and spinal cord lesions. Clinically speaking, irreparable harm could result from a failure to quickly detect and treat these illnesses as well as to correctly determine their underlying causes. This research examines a case report of chorea caused by vitamin B12 deficiency to improve physicians’ diagnostic and differential diagnostic skills for autoimmune gastritis.

## Cases report

The patient was a 66 years-old married male, born in a local rural area and a farmer by occupation. He presented to the Neurology outpatient clinic of our hospital with a chief complaint of involuntary movements of the left hemi-body lasting for 2 days. After initial evaluation, the outpatient physician recommended hospitalization for further diagnostic investigations. The patient and his family consented to the admission.

**FIGURE 1:**
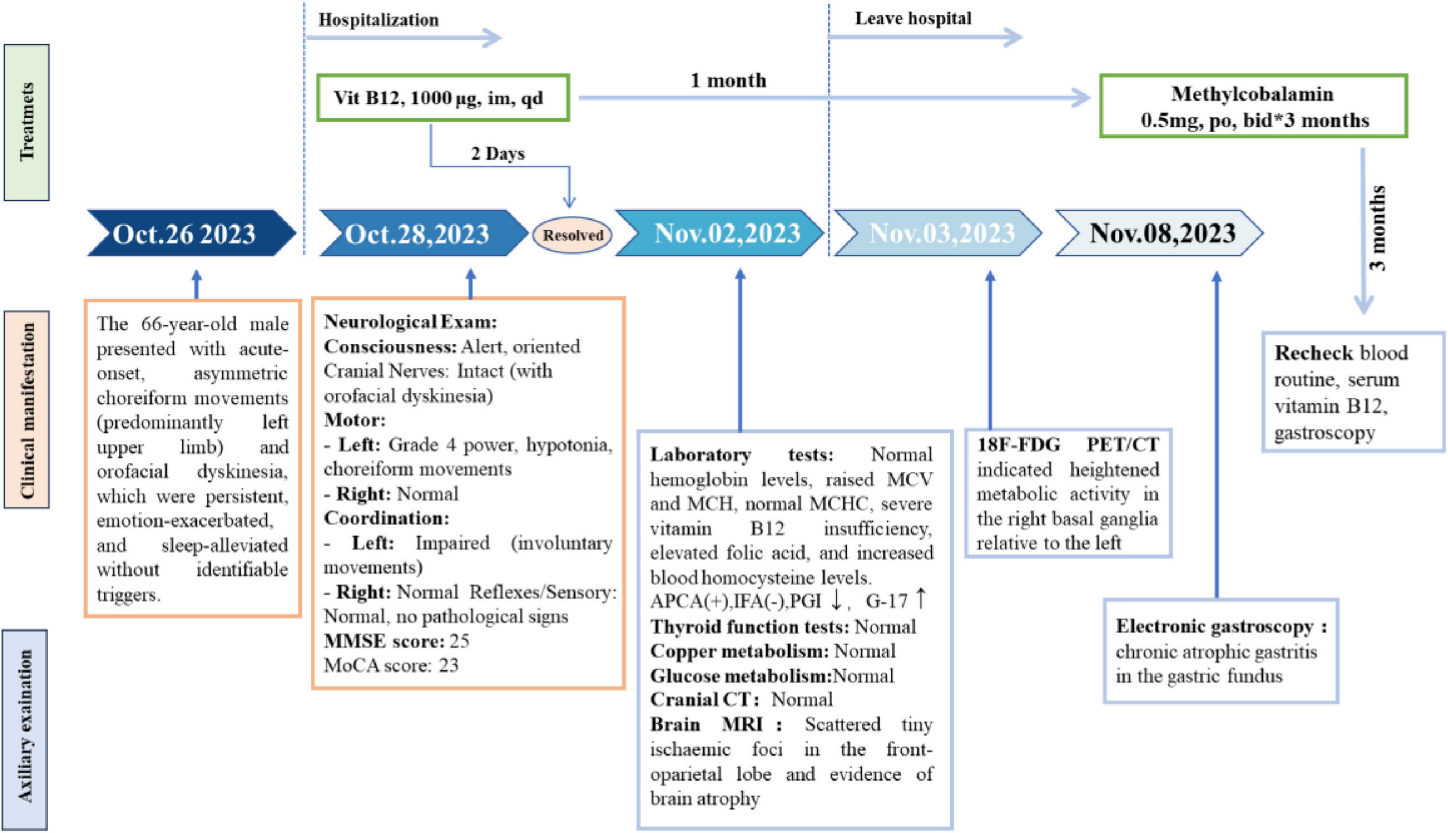
Clinical timeline of symptom evolution, diagnosis, and treatment of the patient.

**FIGURE 2:**
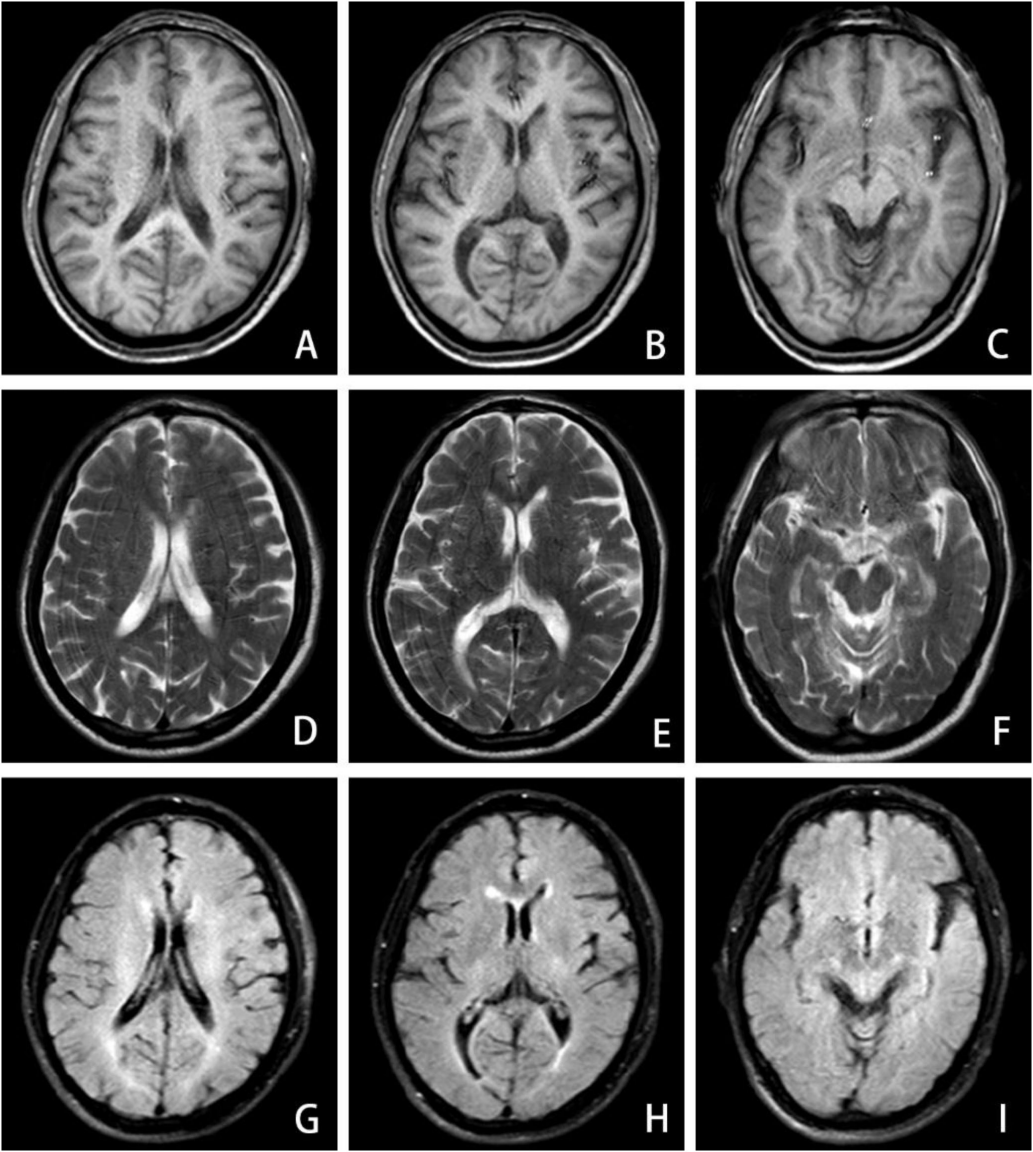
MRI brain imaging exhibits demyelinating-like alterations in the anterior and posterior horns of the lateral ventricles across both cerebral hemispheres, including T1-weighted **(A–C)**, T2-weighted **(D–F)**, and T2-FLAIR **(G–I)** sequences.

**FIGURE 3:**
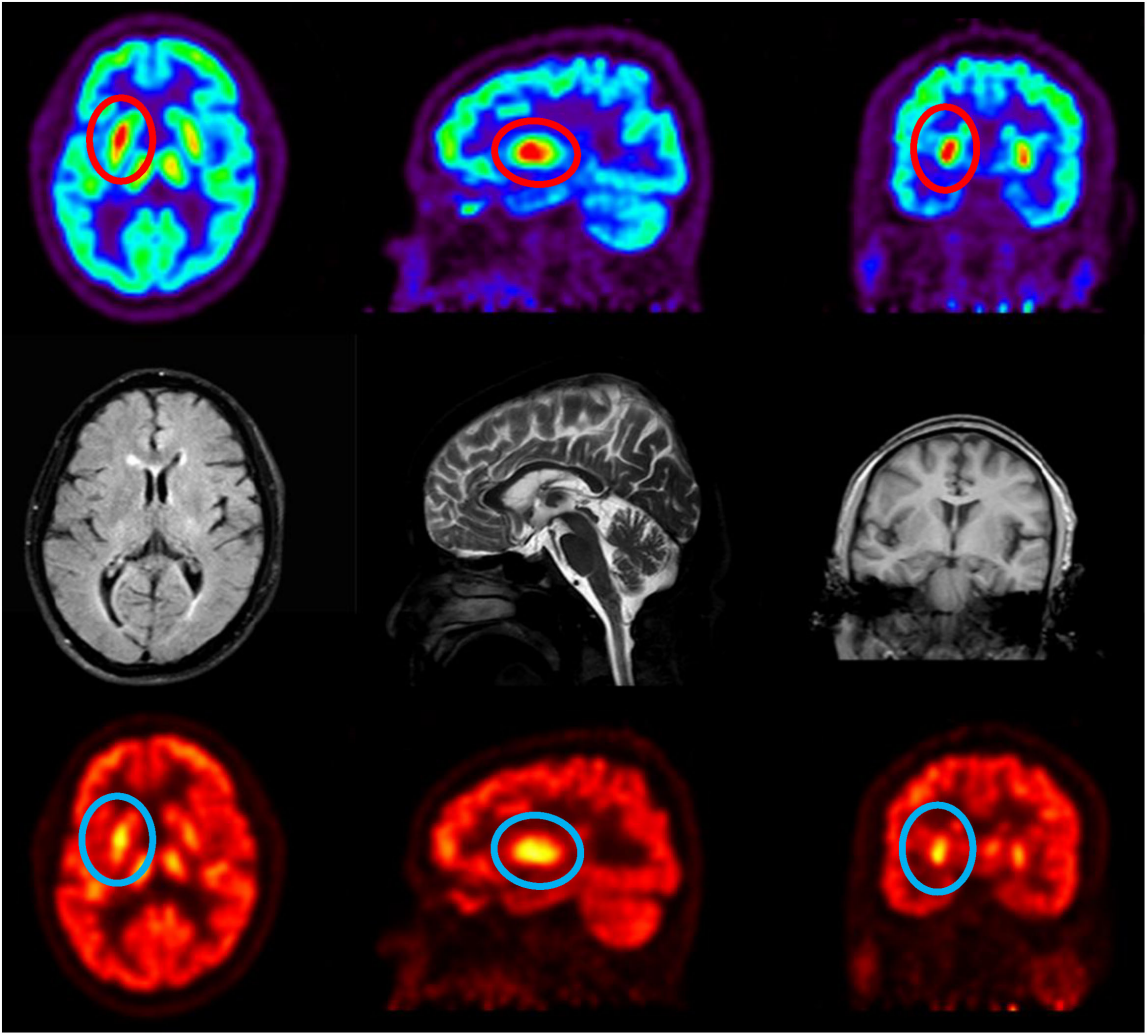
18F-FDG PET/CT reveals metabolic alterations, enhancing diagnostic accuracy. The radioactive tracer 18F-FDG evaluates tissue metabolic activity, revealing markedly elevated metabolism in the right basal ganglia relative to the left.

**FIGURE 4:**
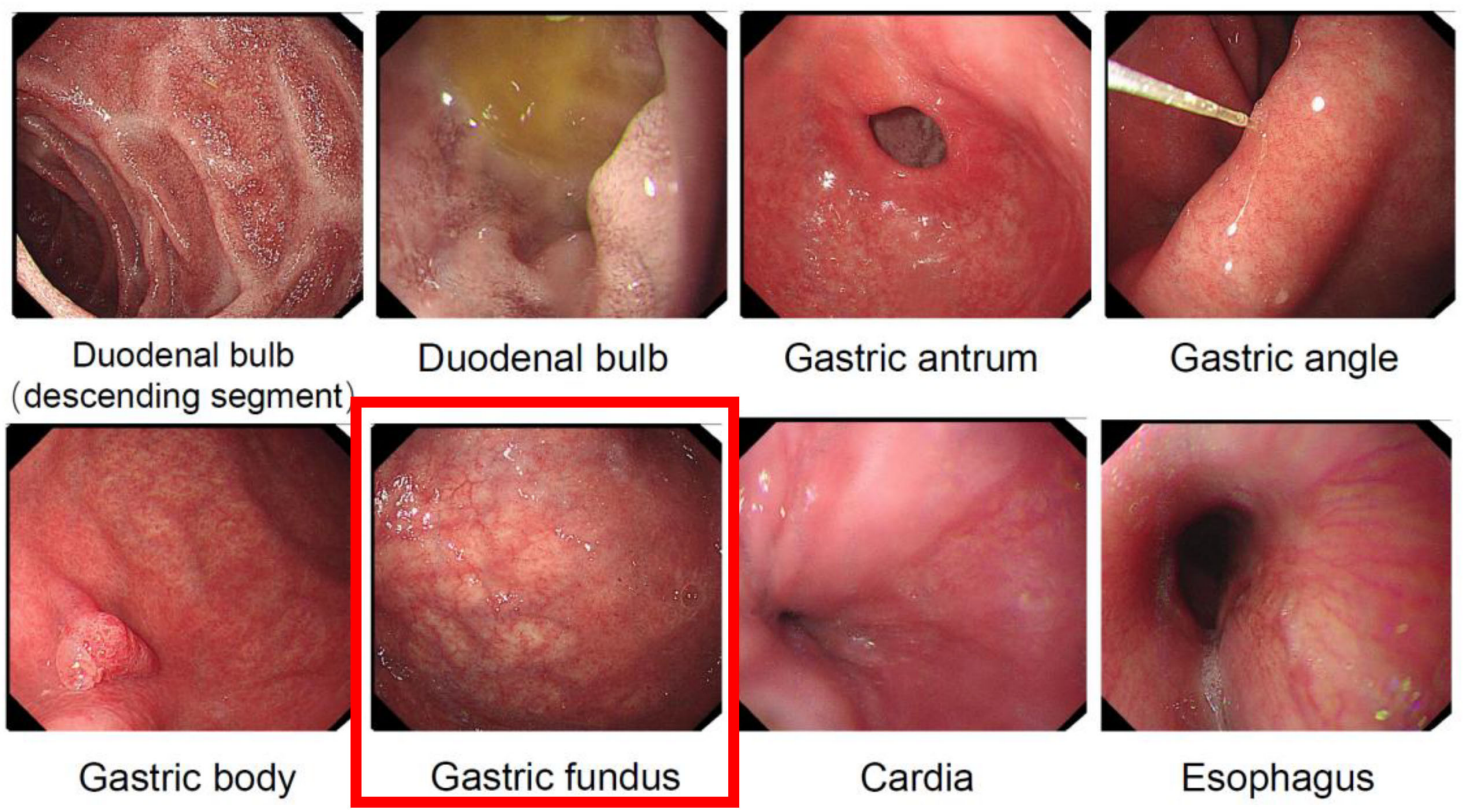
Gastroscopic examination revealing atrophic manifestations of the gastric fundus mucosa. The gastric fundus image demonstrates typical features of gastric fundus mucosal atrophy, with the mucosa appearing pale and thinned, exhibiting prominent vascular patterns, and irregular and erythematous areas visible on the surface.

The patient had suddenly developed strange choreiform spasms in his left leg two days earlier for no apparent reason. The illness was characterized by involuntary stretching and swinging of the left limb, unintended, rhythmless flexion and extension of the left fingers, and more acute symptoms in the left upper limb compared to the left lower limb. Involuntary facial gestures that occur simultaneously include pouting and frowning. The patient had ongoing symptoms that were easily triggered by emotional excitation and that were alleviated in silence and after sleep. When the patient was admitted to the hospital, sensory investigations revealed no abnormalities, clear consciousness, normal orientation and memory, diminished computation, intact cranial nerves, involuntary facial movements, grade 4 left limb muscle strength, decreased muscle tone, uncooperative ataxia on the left side and normal on the right, and a negative cone-bundle sign (Figure 1). The patient was taking antihypertensive medication as directed, had a history of hypertension, was successfully controlling their blood pressure, and had no history of diabetes, ischemic heart disease, mental health disorders, or other systemic diseases. The patient did not use drugs or alcohol, was a vegetarian, and had no family history of genetic problems. Laboratory investigations revealed the following abnormalities: severe vitamin B12 deficiency, elevated folate levels, and increased homocysteine. Additionally, red blood cell indices showed elevated MCV and MCH with normal MCHC and hemoglobin levels. Thyroid function tests, ceruloplasmin, and glucose metabolism were within normal limits (Table 1). The MRI showed signs of brain shrinkage and modest, scattered ischemia foci in the frontoparietal lobe, but the patient’s cranial CT scan came back normal (Figure 2). The right basal ganglia were shown to have more metabolic activity than the left, according to a subsequent 18F-FDG PET/CT investigation (Figure 3). To ascertain the reason of the vitamin B12 shortage, the patient underwent additional testing. According to these tests, the patient had elevated levels of gastrin-17 (G-17), decreased levels of pepsinogen I (PGI), positive IgG antibodies against parietal cells (APCA), negative IgG antibodies against intrinsic factor (IFA), and pathological features of chronic atrophic gastritis in the gastric fundus as shown by gastroscopy (Figure 4). The patient declined a subsequent endoscopic biopsy. The MMSE score was 25, whereas the MoCA score was 23.

**TABLE 1:** Blood test results before treatment.

Test item	Result	Reference values
Erythrocyte, g/L	4.14↓	4.30–5.88*10^12^
Hemoglobin, g/L	149	130–175
Mean corpuscular volume (MCV), fL	103.9↑	82–100
Mean corpuscular hemoglobin (MCH), pg	36↑	27–34
Mean corpuscular hemoglobin concentration (MCHC), g/L	347	316–354
Vitamin B12, pg/mL	0↓	180–914
Ceruloplasmin, g/L	0.17	0.2–0.6
Folate, ng/mL	23.92↑	3.1–19.9
Homocysteine, μmol/l	> 50↑	5.46–16.20
Anti-parietal cell antibody IgG (APCA)	(+)	(−)
Anti-intrinsic factor antibody IgG (IFA)	(−)	(−)
Pepsinogen I (PG I), ng/mL	5.5↓	67.0–200.0
Pepsinogen И (PG И), ng/mL	5.29	< 15.00
Pepsinogen I/И (PG I/И)	1.04↓	≥ 3.00
Gastrin-17 (G-17), pmolL	25.65↑	1.50–7.50

Nursing interventions focused on fall prevention and preventing self/other injury due to involuntary movements, alongside dietary modification to increase vitamin B12 intake; within 2 days of initiating daily 1,000 μg intramuscular vitamin B 12 therapy, the patient achieved complete symptom resolution. Following this rapid response, discharge instructions included continuing therapy at this dosage for one month alongside dietary modifications, with subsequent Neurology outpatient evaluation. At the one-month follow-up, serum vitamin B12 levels were normalized and involuntary movements had resolved completely. Based on this positive response, maintenance therapy was switched to oral mecobalamin supplementation for a further 3 months.

## Discussion

Chorea caused by a vitamin B12 deficiency is not prevalent. The fundamental process is still unclear, and its pathophysiology is complex and poorly understood. However, it is commonly thought to be caused by aberrant signal transmission between the basal ganglia and the frontal motor cortex.

Vitamin B12 (cobalamin), the only vitamin containing a metal ion, plays indispensable roles in human metabolism, including hematopoiesis, neurological function, and nucleic acid synthesis (1, 2). As humans cannot endogenously synthesize this vitamin, dietary intake (primarily from animal sources) and efficient gastrointestinal absorption are critical for maintaining adequate levels. Notably, the body’s substantial B12 reserves and low daily requirements may delay the manifestation of deficiency symptoms for 3–5 years, even with complete intestinal absorption (3). Deficiency arises from insufficient dietary intake, malabsorption (e.g., due to gastric atrophy or autoimmune disorders), impaired cellular transport, or increased metabolic demand (4). The present case underscores these pathways: a long-term vegetarian diet likely contributed to suboptimal intake, while autoimmune atrophic gastritis (confirmed by anti-mural cell antibody positivity and gastroscopic findings) compromised intrinsic factor-mediated absorption, culminating in severe B12 depletion.

Clinically, B12 deficiency manifests across multiple systems. Hematologically, impaired DNA synthesis disrupts erythropoiesis, leading to megaloblastic anemia characterized by fatigue, dizziness, and reduced hemoglobin production (5). Neurologically, demyelination from impaired methionine synthase activity results in subacute combined degeneration, presenting as sensory ataxia, spastic paralysis, and peripheral neuropathy (6). Cognitive disturbances, including memory deficits and confusion, stem from hyperhomocysteinemia-induced neurotoxicity and disrupted neurotransmitter synthesis (7). Psychiatric symptoms such as depression and insomnia further highlight the brain’s vulnerability to B12-dependent metabolic dysregulation (8). Notably, extrapyramidal movement disorders—including chorea, parkinsonism, and dystonia—have been linked to B12 deficiency, though their pathophysiology remains incompletely elucidated.

**FIGURE 5:**
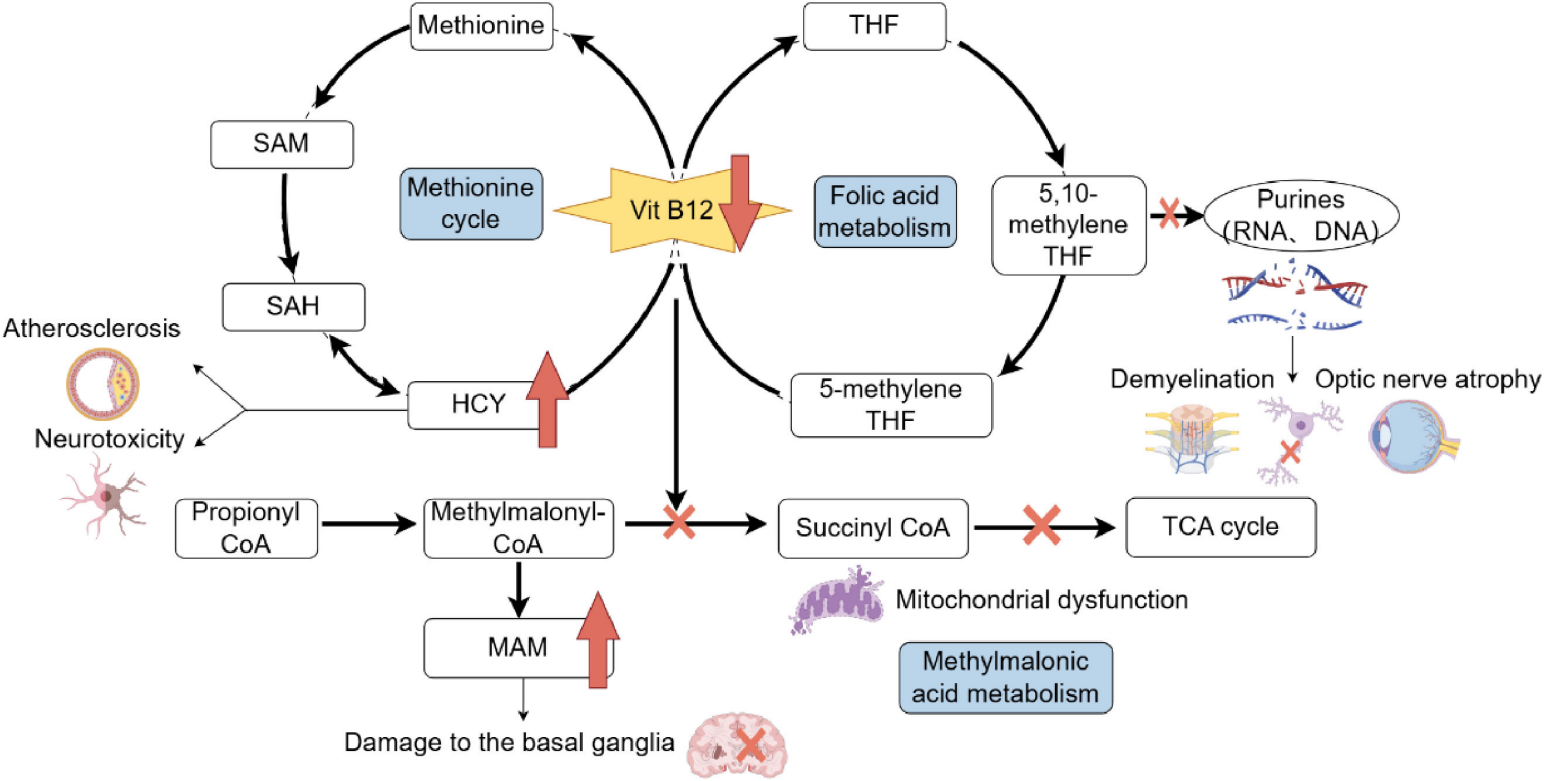
Metabolic pathways impacted by Vitamin B12 deficiency and associated consequences. Vitamin B12 is essential for the methionine cycle, folic acid metabolism, and methylmalonic acid metabolism. Deficiency disrupts these pathways, leading to elevated homocysteine (HCY) levels, which contribute to neurotoxicity and atherosclerosis, and increased methylmalonic acid (MAM), which damages the basal ganglia. Impaired folic acid metabolism affects purine and pyrimidine synthesis, causing RNA and DNA synthesis defects, demyelination, and optic nerve atrophy. Additionally, disruptions in succinyl-CoA synthesis affect the tricarboxylic acid (TCA) cycle, leading to mitochondrial dysfunction. SAH, S-adenosyl homocysteine; SAM, S-adenosylmethionine; HCY, homocysteine; THF, tetrahydrogen folic acid; CoA, coenzyme A; TCA, tricarboxylic acid; MMA, methylmalonic acid.

Emerging evidence implicates two primary mechanisms in B12 deficiency-related neurotoxicity. First, elevated homocysteine levels, resulting from impaired remethylation due to B12’s role as a methyltransferase cofactor, induce oxidative stress, endothelial dysfunction, and excitotoxic NMDA receptor activation, particularly affecting basal ganglia circuits (9–14). This aligns with observations of chorea-like movements following thalamostriatal pathway disruption (15). Second, accumulating 5-methyltetrahydrofolate (5-methyl-THF), a folate metabolite, acts as a dopaminergic agonist, as demonstrated in animal models where its injection induced Huntington’s-like choreiform movements (16, 17). Additionally, methylmalonic acid (MMA) accumulation disrupts mitochondrial energy metabolism, potentially contributing to basal ganglia damage, as seen in methylmalonic acidemia patients with extrapyramidal symptoms (18). Notably, serum homocysteine and MMA levels often precede overt clinical deficits, serving as sensitive biomarkers even when B12 levels are marginally reduced or within normal limits due to compensatory mechanisms (Figure 5). Pallidal hypermetabolism detected by 18F-FDG PET/CT further aids in localizing neuropathology (19). The patient presented with acute hemichorea, a rare extrapyramidal manifestation linked to dopaminergic dysregulation from 5-methyltetrahydrofolate accumulation, rather than classical hematological or neurological deficits. Notably, the rapid resolution of choreiform movements following parenteral B12 supplementation highlights the reversible nature of neuropsychiatric sequelae when intervention precedes irreversible myelin damage. This case has certain inherent limitations: On the one hand, The patient declined a subsequent endoscopic biopsy during follow-up. While a biopsy would have provided valuable diagnostic information, we respected the patient’s decision. On the other hand, The follow-up assessment included monitoring relevant blood parameters; however, a repeat 18F-FDG PET/CT scan was not performed.

Pernicious anemia (PA), serving as an independent risk factor for gastric cancer, is pathologically rooted in autoimmune gastritis (AIG)-induced gastric body atrophy, hypochlorhydria, and intrinsic factor deficiency, culminating in vitamin B12malabsorption (20, 21). Hepatic compensatory B12 reserves may delay anemia onset for years, rendering subacute combined degeneration or metabolic disturbances (e.g., hyperhomocysteinemia) as early warning signals. This case underscores the diagnostic complexity of AIG-associated PA: while prolonged vegetarianism could theoretically contribute to B12 deficiency, the endoscopic finding of extensive gastric mucosal atrophy suggests a dual etiology of malabsorption, necessitating strict differentiation between autoimmune defects and isolated nutritional deficiency. The paradox of profoundly low serum B12 (0 ng/mL) without concurrent anemia further cautions against adherence to traditional diagnostic paradigms, emphasizing the need for vigilance toward atypical presentations.

Moreover, this case exemplifies the critical role of interdisciplinary collaboration. Initially presenting with hemibody involuntary movements, the patient was first evaluated by neurology. Subsequent investigations, including laboratory and imaging assessments, identified severe vitamin B12 deficiency as the causative factor. Further etiological exploration, involving gastroenterology and gastrointestinal surgery evaluations, revealed chronic atrophic gastritis. Hematological analysis subsequently elucidated the correlation between PA and AIG. Thus, the diagnostic journey spanned neurology, gastroenterology, and hematology, highlighting the indispensability of multidisciplinary cooperation in deciphering atypical pathophysiological mechanisms—particularly when neurological symptoms precede classical hematological manifestations. This case urges clinicians to broaden differential diagnostic frameworks to incorporate systemic contributors, thereby fostering enhanced clinical reasoning and holistic patient care.

## Conclusion

This case report describes rare reversible chorea secondary to severe vitamin B12 deficiency, suggesting a novel clinical direction for chorea etiology screening. The diagnostic process exemplifies a multidisciplinary approach. The disease’s multi-organ involvement, evolving symptoms, and often unclear etiology necessitate coordinated cross-specialty diagnostics and management. This case also underscores that autoimmune gastritis serves as a bridge between immunology and gastroenterology. When AIG induces severe vitamin B12 deficiency, it can manifest with neurological sequelae, including cerebral involvement. Therefore, broadening diagnostic paradigms and enhancing interdisciplinary collaboration are crucial for timely recognition and effective management.

## Data Availability

The datasets presented in this article are not readily available because of ethical and privacy restrictions. Requests to access the datasets should be directed to the corresponding authors.
